# Expression of a recombinant hybrid antimicrobial peptide magainin II-cecropin B in the mycelium of the medicinal fungus *Cordyceps militaris* and its validation in mice

**DOI:** 10.1186/s12934-018-0865-3

**Published:** 2018-02-05

**Authors:** Min Zhang, Yuanlong Shan, Hongtao Gao, Bin Wang, Xin Liu, Yuanyuan Dong, Xiuming Liu, Na Yao, Yonggang Zhou, Xiaowei Li, Haiyan Li

**Affiliations:** College of Life Sciences, Engineering Research Center of the Chinese Ministry of Education for Bioreactor and Pharmaceutical Development, Jilin Agricultural University, Changchun, 130118 Jilin China

**Keywords:** Hybrid antimicrobial peptide, *Cordyceps militaris*, Recombinant expression, *Escherichia coli* (ATCC 25922), Immunomodulatory, Feed additive

## Abstract

**Background:**

Antibiotic residues can cause antibiotic resistance in livestock and their food safety-related issues have increased the consumer demand for products lacking these residues. Hence, developing safe and effective antibiotic alternatives is important to the animal feed industry. With their strong antibacterial actions, antimicrobial peptides have potential as antibiotic alternatives.

**Results:**

We investigated the antibacterial and immunomodulatory activities and the mechanisms of action of an antimicrobial peptide. The hybrid antimicrobial peptide magainin II-cecropin B (Mag II-CB) gene was transformed into the medicinal *Cordyceps militaris* fungus. Recombinant Mag II-CB exhibited broad-spectrum antibacterial activity in vitro and its antibacterial and immunomodulatory functions were evaluated in BALB/c mice infected with *Escherichia coli* (ATCC 25922). Histologically, Mag II-CB ameliorated *E. coli*-related intestinal damage and maintained the integrity of the intestinal mucosal barrier by up-regulating tight junction proteins (zonula occludens-1, claudin-1 and occludin). The intestinal microbial flora was positively modulated in the Mag II-CB-treated mice infected with *E. coli*. Mag II-CB treatment also supported immune functioning in the mice by regulating their plasma immunoglobulin and ileum secreted immunoglobulin A levels, by attenuating their pro-inflammatory cytokine levels, and by elevating their anti-inflammatory cytokines levels. Moreover, directly feeding the infected mice with the *C. militaris* mycelium producing Mag II-CB further proofed the antibacterial and immunomodulatory functions of recombinant hybrid antimicrobial peptide.

**Conclusion:**

Our findings suggest that both purified recombinant AMPs and *C. militaris* mycelium producing AMPs display antibacterial and immunomodulatory activities in mice. And *C. militaris* producing AMPs has the potential to become a substitute to antibiotics as a feed additive for livestock in future.

**Electronic supplementary material:**

The online version of this article (10.1186/s12934-018-0865-3) contains supplementary material, which is available to authorized users.

## Background

Antibiotics with broad-spectrum antibacterial and growth promoting activities are used widely in livestock and poultry production as feed additives for maintaining the health and productivity of these animals [1]. In 2010, the global consumption of antibiotics for food animal production was estimated at 63,151 tons [2]. However, the presence of antibiotic residues in food is recognized as a problem in different parts of the world, and public health concerns exist regarding antibiotic resistance, toxicity and teratogenicity. Therefore, there is an urgent need to develop alternatives to antibiotics for use as feed additives for livestock [3].

Comprising mainly cationic and amphipathic polypeptides, antimicrobial peptides (AMPs), are able to target pathogens by stopping them traversing external barriers and destroying them internally [4, 5]. Compared with traditional antibiotics, AMPs have broad spectrum characteristics [6], enabling them to effectively target fungi, parasites, viruses and tumor cells [7, 8], which makes them attractive as pharmaceuticals and feed additives, for example [9, 10]. More than 2300 AMP types have been found in animals, plants and bacteria [4, 11]. Among them, magainins, which are 21–27 amino acid residues long and are isolated from *Xenopus laevis* skin [12], contain the α-helical secondary structures that form the transmembrane ion channels needed for their antibacterial effects [13]. Within the magainin family, magainin II (Mag II) exerts its lethal effects by causing membrane lysis in Gram-positive and Gram-negative bacteria, as well as in tumor cells [14–16]. Cecropins, a natural peptide of the *Cecropia moth* [17], contain 31–39 amino acid residues and adopt an α-helical structure on interaction with bacterial membranes to form ion channels [18]. Among the cecropin family, cecropin B (CB) is considered a good candidate molecule for enhancing resistance to bacterial diseases because of its very strong antibacterial activity against Gram-negative bacteria [19].

In addition to the naturally-occurring AMPs, many recombinant AMPs have been designed to achieve increased stability and specificity [20–22]. These recombinant AMPs, which have been produced in various expression systems and exhibit different properties, have been used to enhance pathogen resistance in plants and enhance immunomodulatory functions in animals [21, 23–26]. Coca et al. [23] reported that cecropin A protein, when expressed in recombinant rice plants, was biologically active against the rice blast fungus *Magnaporthe grisea*. In addition, when CB was expressed in recombinant tomato plants it was effective against two major bacterial tomato diseases [24]. Rivero et al. [25] also found that expressing AMPs in potato plants increased their resistance to bacterial and fungal pathogens.

Hybrid peptides are antimicrobial peptides that are fused to other AMPs or functional proteins to provide bifunctional properties. Some studies [27, 28] have indicated that cecropin A (1-8)-Mag II (1-12) (CA-MA), a hybrid antimicrobial peptide, exhibits higher antibacterial activity than that of cecropin A or Mag II. However, the properties and functions of hybrid antimicrobial peptide magainin II-CB have not been investigated.

Previous studies have shown that the medicinal components of the *Ascomycetes* fungus *Cordyceps militaris* (i.e., cordycepin, and polysaccharides) have immunomodulatory functions [29] and anti-tumor and anti-oxidative activities [30–32]; hence, this fungus has been used in the pharmaceutical field and in health promoting foods, and its other potential applications are under investigation [33, 34]. However, *C. militaris* has not yet been reported as a vehicle for AMP production. Therefore, further studies are required to explore whether *C. militaris* mycelium is a candidate host for the development of AMPs because of its various medicinal ingredients and its short growth cycle, good safety profile and its ability to be taken orally. Based on these potential advantages, in the present study, we aimed to express magainin II-CB (Mag II-CB), a hybrid antimicrobial peptide, in *C. militaris* mycelium. The antibacterial function of the AMPs was evaluated in vitro using antibacterial assays, and in vivo after their administration to BALB/c mice infected with *Escherichia coli* (*E. coli*, ATCC 25922). We also investigated the immunomodulatory activities of the recombinant hybrid antimicrobial peptide and *C. militaris* producing AMPs, and provide here preliminary scientific evidence for the future production of *C. militaris* mycelium producing AMPs as potential feed additives for livestock.

## Results

### Construction of the hybrid antimicrobial peptide gene Mag II-CB and the single CB gene expression vectors and *C. militaris* was transformed with expression constructs

**Fig. 1:**
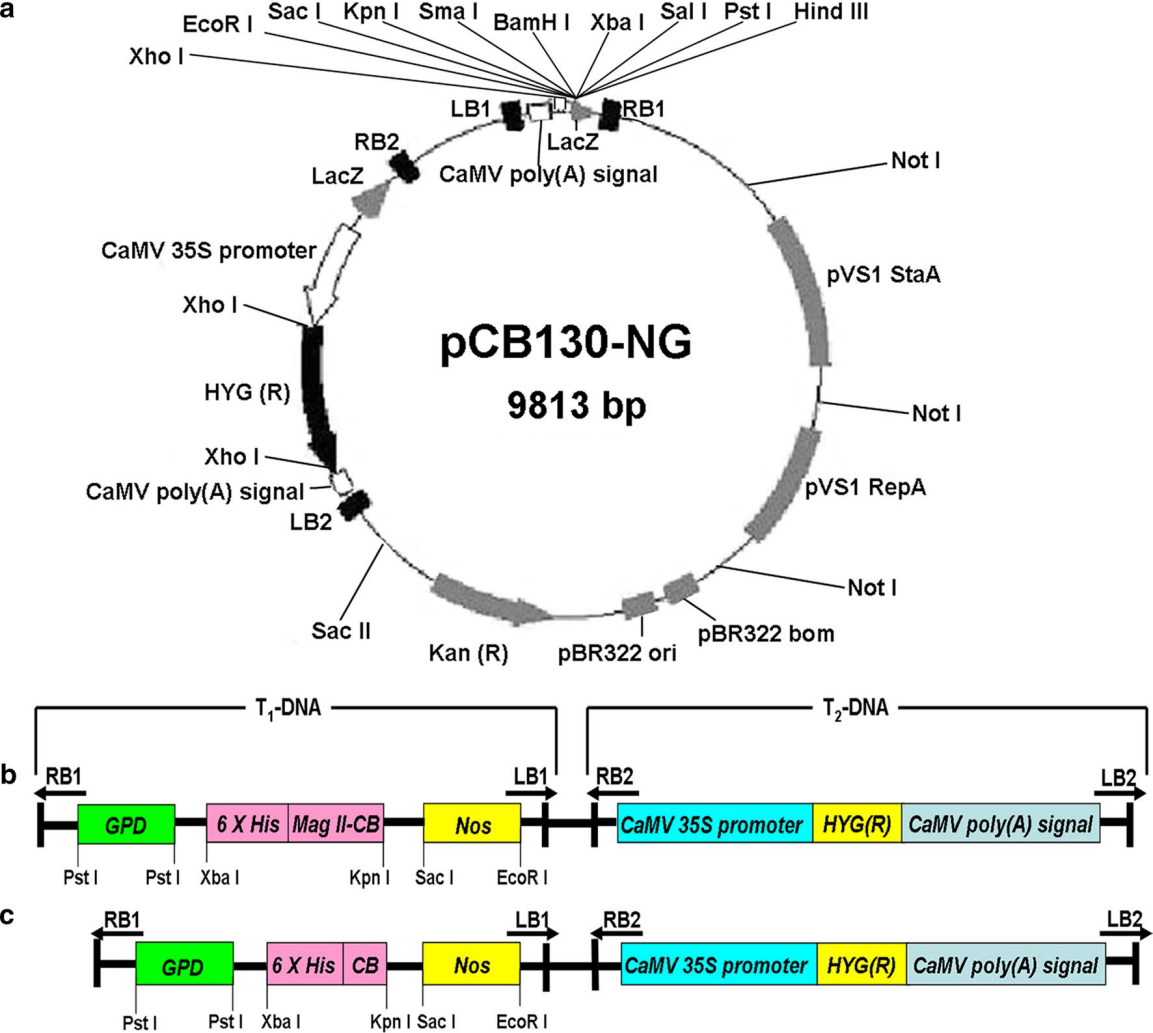
Expression vector construction. The binary T-DNA expression vector pCB130-NG was used in this study (**a**). In T_1_-DNA region, expression of the target gene was driven by the *GPD* (glyceraldehyde-3-phophate dehydrogenase) promoter and the *Nos* (nopaline synthase gene) terminator. In T_2_-DNA region, the *HYG* gene encoding hygromycin B served as a selectable marker for transformation, and its expression was driven by the *Cauliflower mosaic virus* (CaMV) 35S promoter and the CaMV poly(A) signal. LB and RB are the left and right border regions, respectively. Schematic diagram of the recombinant plasmids pCB130-Mag II-CB (**b**) and pCB130-CB (**c**) used to transform *Cordyceps militaris* mycelia

The binary T-DNA expression vector, which contains the hybrid antimicrobial peptide gene Mag II-CB, was successfully constructed. As a control with which to compare and further investigate the functions of the hybrid antimicrobial peptide, the pCB130-CB expression vector containing the single antimicrobial CB peptide was also constructed (Fig. 1). *Agrobacterium tumefaciens* was then separately transformed with the pCB130-Mag II-CB and pCB130-CB plasmids and approximately 30–60 transformants per 1 × 10^5^ protoplasts were recorded after their cocultivation. To determine whether the Mag II-CB and CB genes had been transferred to *C. militaris*, PCR was conducted on the genomic DNA from the transformed *C. militaris* as the template. The gel electrophoresis image shows that 10 clones of the Mag II-CB transformed *C. militaris* (Additional file 1: Figure S1) and 12 clones of the CB transformed *C. militaris* (Additional file 1: Figure S1) were obtained respectively, and these clones were used for further analysis.

### Expression, purification and quantification of Mag II-CB and CB from transformed *C. militaris*

**Fig. 2:**
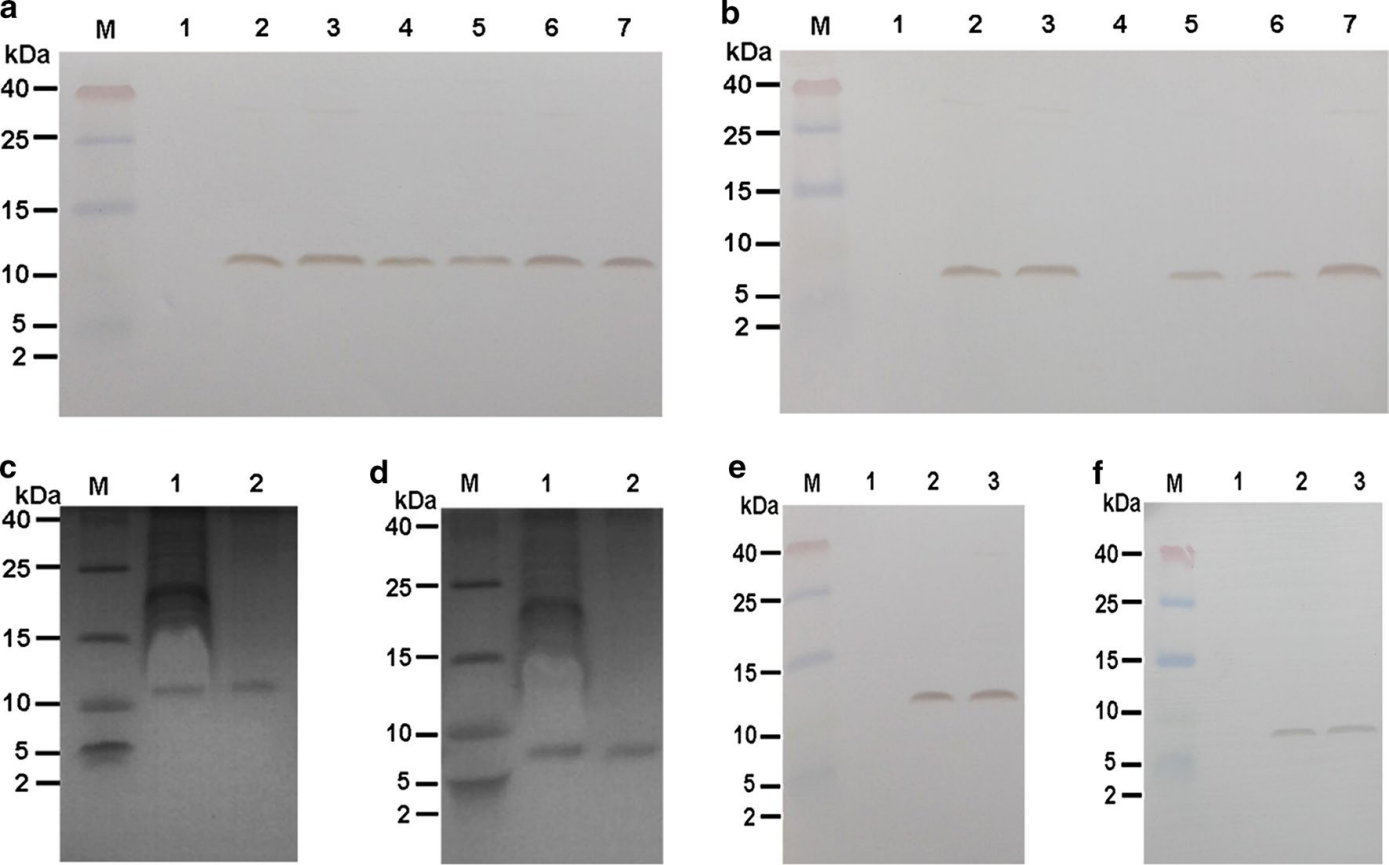
Western blotting was performed with the total protein isolated from WT and transformed Mag II-CB (**a**) and CB (**b**) *C. militaris*. Lane 1: WT *C. militaris*. Lanes 2–7: Transformed Mag II-CB (**a**) and CB (**b**) *C. militaris*. M denotes the protein marker. Coomassie blue-stained SDS-PAGE products showing Mag II-CB (**c**) and CB (**d**) proteins expressed by transformed *C. militaris* (lane 1) and the purified samples (lane 2). Western blotting analysis of the Mag II-CB (**e**) and CB (**f**) protein in WT *C. militaris* (lane 1), transformed *C. militaris* (lane 2) and the purified samples (lane 3)

To confirm that the Mag II-CB and CB antimicrobial peptides were expressed in *C. militaris*, the total protein content extracted from wild-type (WT) *C. militaris* and different clones of transformed *C. militaris* was analyzed by western blotting (Fig. 2a, b). The Mag II-CB and CB protein bands were approximately 10.8 and 6.2 kDa, respectively, but no visible protein bands were detected in the WT *C. militaris* extract at these positions. The ELISA assays showed that the highest contents for Mag II-CB and CB were 4.5 mg and 3.38 mg/g of the freeze-dried mycelium powders, respectively. Therefore, the cells lines with the highest Mag II-CB and CB expression levels were used for further experiments. The His-tagged proteins were purified and analyzed by Tricine-SDS-PAGE and western blots. The purified Mag II-CB and CB proteins each had only one band on the gels (Fig. 2c–f), and the molecular weights of the bands were consistent with the results shown in Fig. 2a, b. The purification yields for Mag II-CB and CB were 3.86 mg and 2.95 mg/g of the freeze-dried mycelium powders and the purities of the Mag II-CB and CB proteins were 82.6 and 80.4%, respectively, as judged by ELISAs, thereby confirming that the recombinant antimicrobial peptides Mag II-CB and CB had been successfully expressed in *C. militaris* and could be used for further studies.

### Antibacterial activity of the Mag II-CB recombinant hybrid antimicrobial peptide

**Fig. 3:**
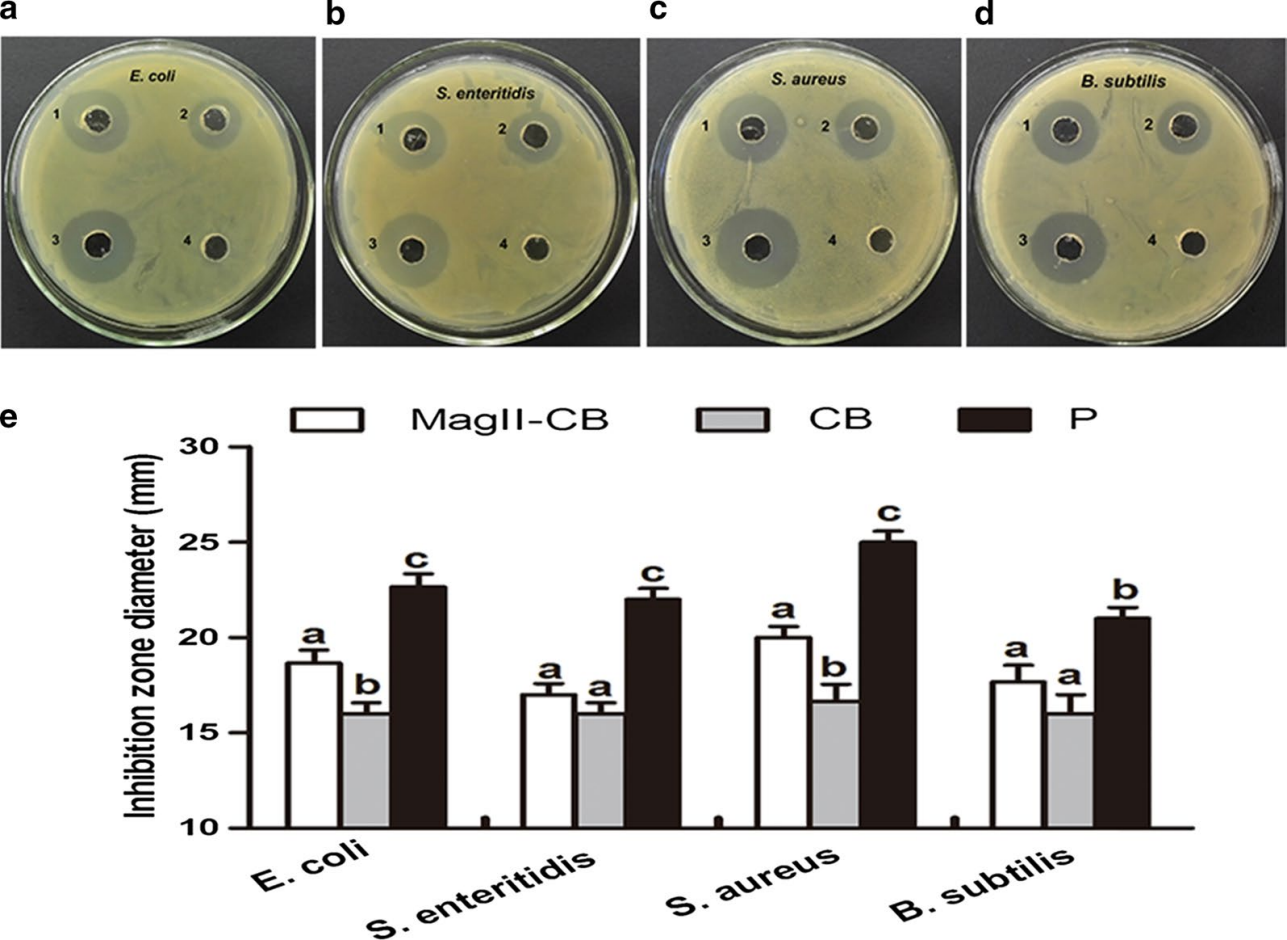
Analysis of the antibacterial activities of Mag II-CB and CB against *E. coli* (**a**), *S. enteritidis* (**b**), *S. aureus* (**c**) and *B. subtilis* (**d**). (1): recombinant Mag II-CB hybrid antibacterial peptide; (2): recombinant single antibacterial CB peptide; (3): streptomycin positive control; (4): total protein extracted from WT *C. militaris* served as a negative control. The diameter of the inhibition zone was measured (**e**). The final values were the mean ± SE, and values without the same letters are significantly different (*P* < 0.05)

To investigate the antibacterial activity of the Mag II-CB hybrid antimicrobial peptide in vitro, the diameter of the agar plate bacterial diffusion clearance zone was measured. A significant inhibition zone for both Gram-negative and Gram-positive bacteria was observed in the group treated with the Mag II-CB peptide and in the streptomycin-positive controls, but not in the WT control treated with the *C. militaris* extract (Fig. 3). Concurrently, the cells treated with the single antimicrobial CB peptide exhibited smaller inhibition zones for both Gram-negative and Gram-positive bacteria than those observed after Mag II-CB treatment.

To determine the minimal inhibitory concentrations (MICs) of Mag II-CB and CB, different concentrations of them were assayed against Gram-negative and Gram-positive bacteria (Table 1). The Mag II-CB hybrid antimicrobial peptide was more effective at inhibiting the growth of *S. aureus*, *E. coli* and *B. subtilis* (with MICs of 2, 4 and 4 μg/mL, respectively) than the single antimicrobial CB peptide. Mag II-CB and CB inhibited most of the Gram-negative and Gram-positive bacteria with low MICs, but CB was less effective at inhibiting the growth of *S. xylosus.* Overall, the recombinant Mag II-CB from the transformed *C. militaris* exhibited effective antibacterial activities in terms of its ability to inhibit Gram-negative and Gram-positive bacteria.

**Table 1:** MICs of antibacterial peptides Mag II-CB and CB against eight bacterial strains

Bacteria strains	MIC (µM)	
	Mag II-CB	CB
*Escherichia coli*	4	8
*Salmonella enteritidis*	8	8
*Enterobacter aerogenes*	8	8
*Proteus mirabilis*	16	16
*Staphylococcus aureus*	2	4
*Bacillus subtilis*	4	8
*Enterococcus faecalis*	8	16
*Staphylococcus xylosus*	32	> 128

### The effects of Mag II-CB on mouse intestinal microbial flora and intestine histology

**Fig. 4:**
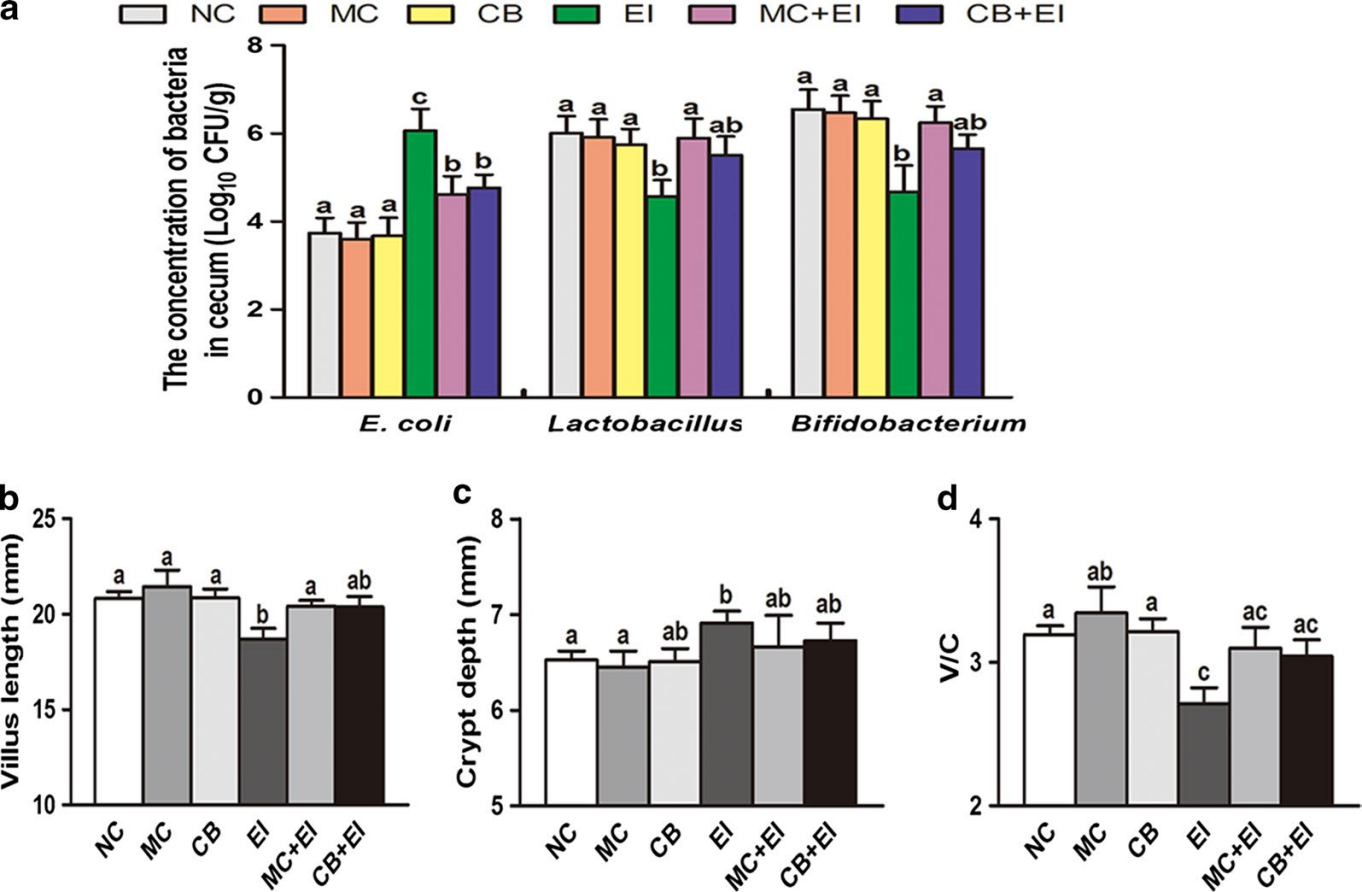
The effects of Mag II-CB and CB treatment on the intestinal microbial flora, the intestine villi, and the intestinal crypts of the experimental mice. The numbers of *E. coli*, *Lactobacillus* and *Bifidobacterium* in the cecum (**a**) were counted using the dilution plate colony counting method. The villus length (**b**), crypt depth (**c**) and V/C value (**d**) in the mice were determined. The final values were the mean ± SE, and values without the same letters are significantly different (*P* < 0.05)

**Fig. 5:**
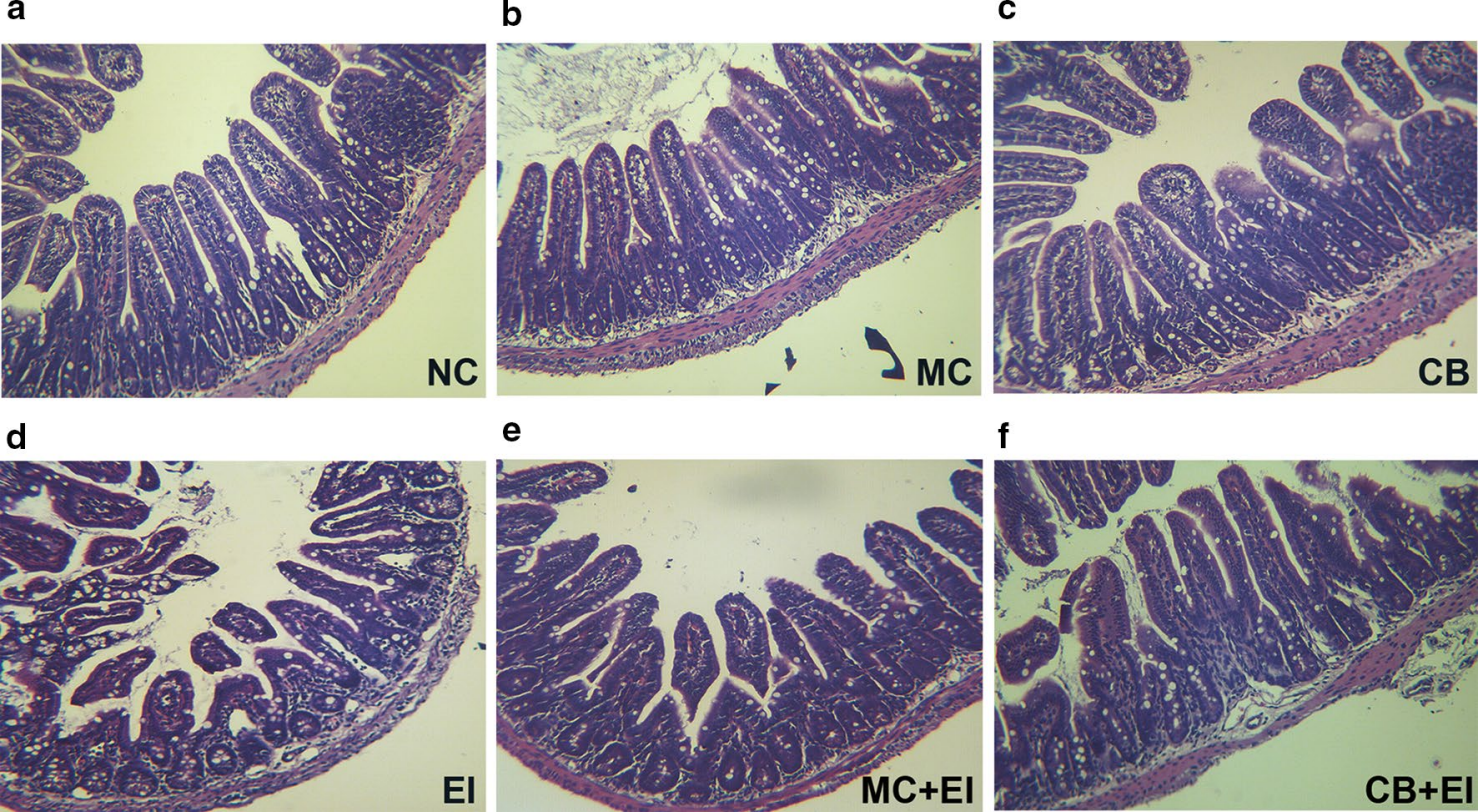
The effects of Mag II-CB and CB treatment in the mice, as determined by intestinal histology. Representative images for the different groups are shown at ×40 magnification. **a** Normal control (NC); **b** recombinant Mag II-CB (MC); **c** recombinant CB (CB); **d** *Escherichia coli* (ATCC 25922) (EI); **e** *E. coli*-infected mice treated with Mag II-CB (MC + EI); **f** *E. coli*-infected mice treated with CB (CB + EI)

Based on the antibacterial effects of the Mag II-CB recombinant hybrid antimicrobial peptide in vitro, we used mice that had been infected with *E. coli* (ATCC 25922) to further investigate its antibacterial activities in vivo. To count the numbers of *E. coli*, *Lactobacillus* and *Bifidobacterium* in the cecal contents of the mice, we used the dilution plate colony counting method. As shown in Fig. 4a, Mag II-CB significantly prevented the *E. coli*-mediated increase in cecal *E. coli* numbers and prevented a decrease in *Lactobacillus* and *Bifidobacterium* numbers. The single antimicrobial CB peptide also markedly inhibited the *E. coli*-induced increase in cecal *E. coli* numbers, whereas it was not significant in increasing the cecal *Lactobacillus* and *Bifidobacterium* numbers compared with EI group mice. To investigate the effects of the Mag II-CB hybrid antimicrobial peptide on the mouse intestine, we performed a histological analysis on the mouse ileum. As shown in Fig. 5, mucous layer destruction and atrophied villi were apparent after infection with *E. coli*; however, the level of damage was reduced in the Mag II-CB-treatment group. Moreover, compared with the *E. coli* infected group, longer villus, decreased crypt depth and an elevated V/C value were observed for the Mag II-CB treatment group, but the effects of the single antimicrobial CB peptide were less than those of the Mag II-CB group (Fig. 4b–d). These results indicate that the Mag II-CB hybrid antimicrobial peptide had anti-bacterial properties in the mice, a finding consistent with the results of our in vitro study.

### Mag II-CB hybrid antimicrobial peptide treatment ameliorates damage to the intestinal mucosal barrier in mice

**Fig. 6:**
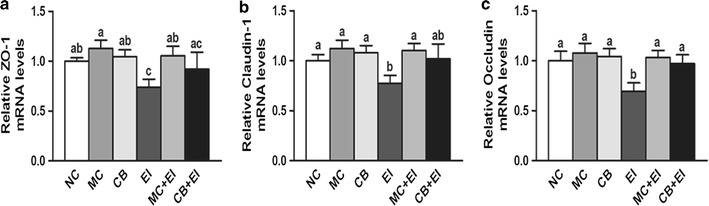
The effects of Mag II-CB and CB treatment on tight junction proteins in mice. The Ileum mRNA levels of ZO-1 (**a**), claudin-1 (**b**) and occludin (**c**) were analyzed by real-time RT-PCR. The final values were the mean ± SE, and values without the same letters are significantly different (*P* < 0.05)

A critical component of the intestinal mucosal barrier is the intercellular junctional complexes between adjacent intestinal epithelial cells, which consist of tight junctions, adherens junctions, desmosomes and gap junctions [35]. Among them, tight junctions play a critical role in regulating the permeability of the intestinal mucosal barrier and resisting the penetration of endotoxins and exogenous pathogens. Zonula occludens-1 (ZO-1), claudin-1, and occludin are three key proteins in tight junctions. As shown in Fig. 6a–c, *E. coli* infection significantly decreased ZO-1, claudin-1 and occludin mRNA expression, as compared with their expression levels in the NC mice. However, treatment with the Mag II-CB hybrid antimicrobial peptide markedly inhibited the *E. coli*-induced tight junction protein decrease, whereas the effect of the single antimicrobial CB peptide was less pronounced than that of Mag II-CB peptide, indicating that the hybrid antimicrobial peptide plays a positive modulating role in intestinal tight junctions and barrier functionality.

### Immunomodulatory function of the Mag II-CB hybrid antimicrobial peptide in mice

**Fig. 7:**
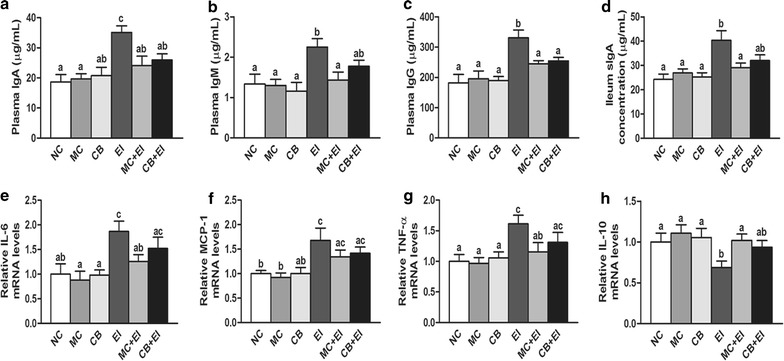
Immunomodulatory function of Mag II-CB and CB in mice and intestinal inflammatory cytokine levels. Plasma IgA (**a**), IgM (**b**), IgG (**c**) and ileum sIgA (**d**) levels were measured using ELISA kits. The ileum-specific inflammatory cytokine levels for IL-6 (**e**), MCP-1 (**f**), TNF-α (**g**) and IL-10 (**h**) were also measured by real-time RT-PCR. The final values were the mean ± SE, and values without the same letters are significantly different (*P* < 0.05)

To assess the immunomodulatory effects of the Mag II-CB hybrid antimicrobial peptide, the levels of innate immune effector molecules (i.e., immunoglobulin IgA, IgM and IgG), were assayed. We found that the mice infected with *E. coli* had significantly increased plasma IgA, IgM and IgG levels compared with the NC mice. However, Mag II-CB treatment markedly attenuated the levels of all three plasma immunoglobulins, whereas the single antimicrobial CB peptide markedly attenuated the levels of IgA and IgG alone (Fig. 7a–c). In addition, Mag II-CB treatment of the infected mice markedly improved their ileal sIgA levels (Fig. 7d).

In innate and adaptive immunity, AMPs can act as effector molecules to modulate pro-inflammatory and anti-inflammatory responses [36]. Therefore, we measured the levels of intestinal inflammatory cytokines to determine the effects of the Mag II-CB hybrid antimicrobial peptide in the mice. As shown in Fig. 7e–g, *E. coli* infection markedly increased the mRNA levels of the pro-inflammatory cytokines interleukin (IL)-6, monocyte chemotactic protein-1 (MCP-1) and tumor necrosis factor alpha (TNF-α), but such increases were prevented by Mag II-CB and CB peptide treatment, with the Mag II-CB peptide being better at prevention than the single CB antimicrobial peptide. Moreover, Mag II-CB treatment substantially increased expression of the anti-inflammatory IL-10 cytokine (Fig. 7h). In summary, the Mag II-CB hybrid antimicrobial peptide was found to protect mice from bacteria not only by its direct antibacterial activities, but also by its immunomodulatory functions.

### Antibacterial and immunomodulatory functions of *C. militaris* producing Mag II-CB in mice

**Fig. 8:**
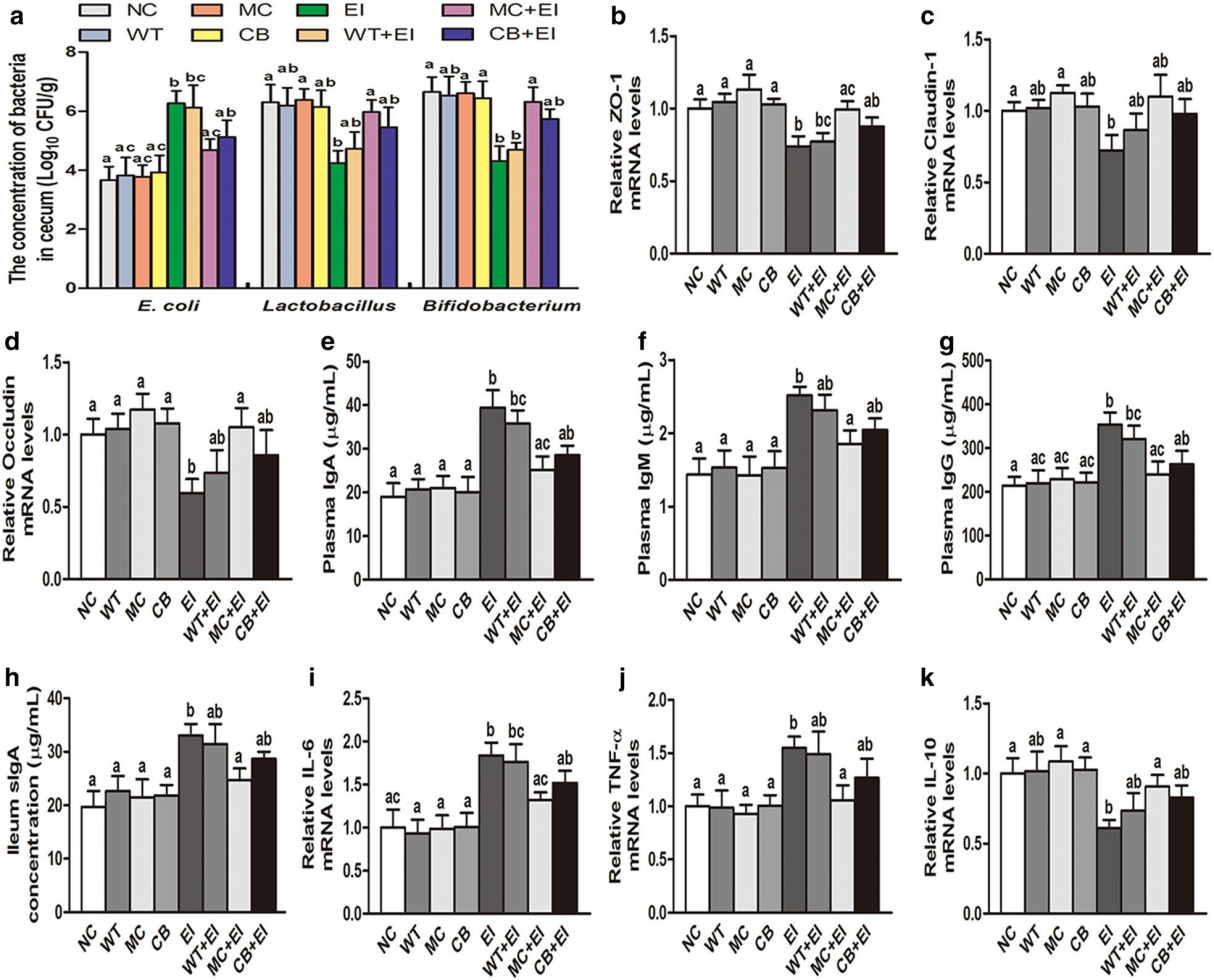
Antibacterial and immunomodulatory functions of the *C. militaris* producing Mag II-CB and CB in mice. The numbers of *E. coli*, Lactobacillus and Bifidobacterium in the cecum (**a**) were counted. The Ileum mRNA levels of ZO-1 (**b**), claudin-1 (**c**) and occludin (**d**) were analyzed by real-time RT-PCR. Plasma IgA (**e**), IgM (**f**), IgG (**g**) and ileum sIgA (**h**) levels were measured using ELISA kits. The ileum-specific inflammatory cytokine levels for IL-6 (**i**), TNF-α (**j**) and IL-10 (**k**) were also measured by real-time RT-PCR. The final values were the mean ± SE, and values without the same letters are significantly different (P < 0.05)

**Fig. 9:**
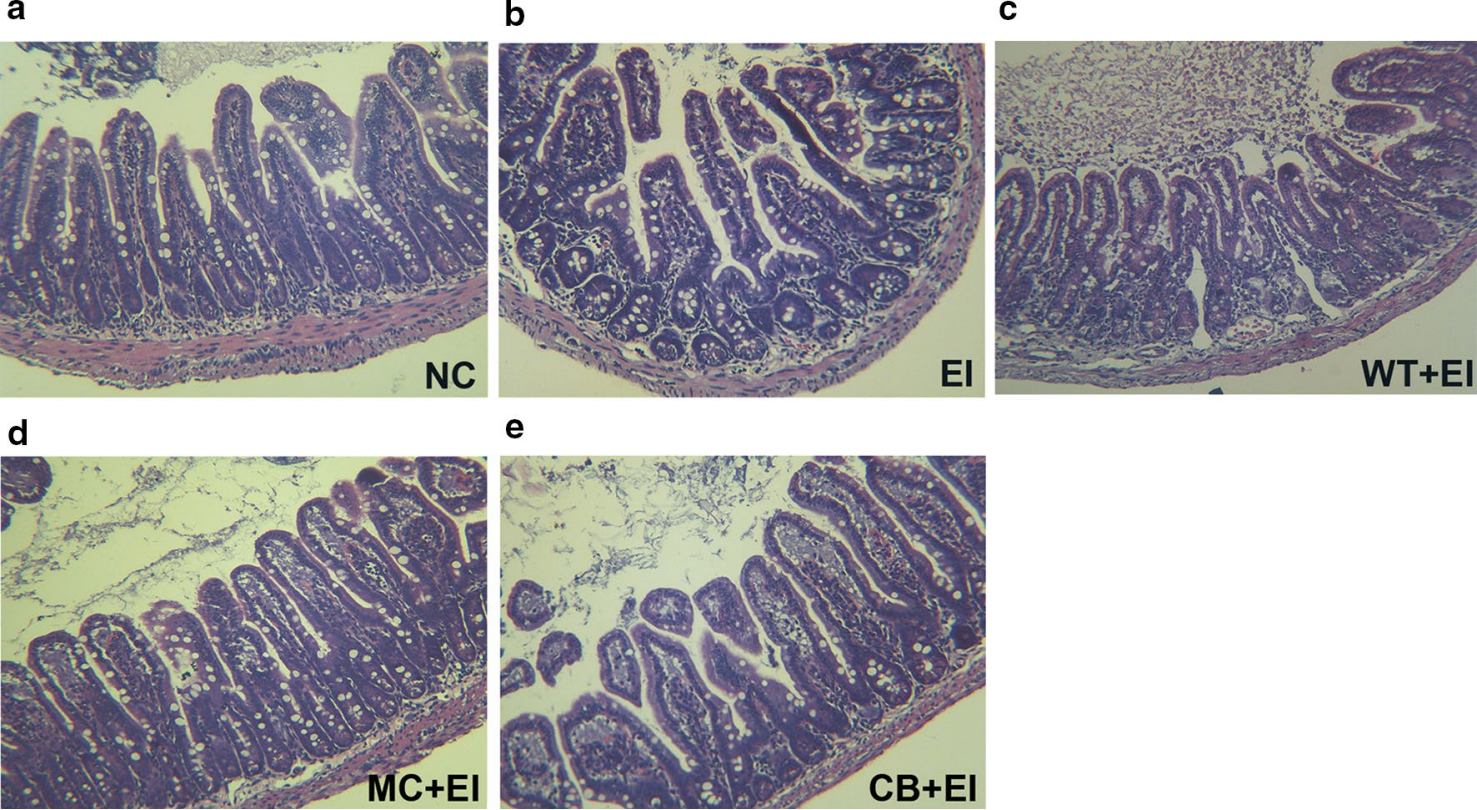
The effects of the *C. militaris* producing Mag II-CB and CB in the mice, as determined by intestinal histology. Representative images for the different groups are shown at ×40 magnification. **a** normal control (NC); **b** *E. coli* (ATCC 25922) (EI); **c** *E. coli*-infected mice administered with WT *C. militaris* mycelium powders (WT + EI); **d** *E. coli*-infected mice administered with the *C. militaris* producing Mag II-CB mycelium powders (MC + EI); **e** *E. coli*-infected mice administered with the *C. militaris* producing CB mycelium powders (CB + EI)

Based on the antibacterial and immunomodulatory effects of the Mag II-CB recombinant hybrid antimicrobial peptide in vivo, we directly fed the mice that had been infected with *E. coli* (ATCC 25922) with the *C. militaris* mycelium producing AMPs to further investigate its antibacterial and immunomodulatory activities. Consistent with the results of Mag II-CB’s antibacterial effects in BALB/c mice infected with *E. coli*, directly feeding the mice with the *C. militaris* mycelium producing Mag II-CB significantly prevented the *E. coli*-mediated increase in cecal *E. coli* numbers and prevented a decrease in *Lactobacillus* and *Bifidobacterium* numbers, but the effect of the *C. militaris* mycelium producing CB was less than that of the Mag II-CB group (Fig. 8a). As shown in Fig. 8b–d, *E. coli* infection significantly decreased tight junction protein ZO-1, claudin-1 and occludin mRNA expression compared with NC mice, however, treatment with the *C. militaris* mycelium producing Mag II-CB markedly inhibited the *E. coli*-induced ZO-1 and occluding decrease, and there was a trend toward a decrease in claudin-1 mRNA expression, whereas the effect of the *C. militaris* mycelium producing CB was less pronounced than that of Mag II-CB peptide. Moreover, the *C. militaris* mycelium producing Mag II-CB reduced the level of damage in mucous layer destruction and atrophied villi after infection with *E. coli* (Fig. 9).

On the other hand, to assess the immunomodulatory effects of the *C. militaris* mycelium producing AMPs, we measured the levels of plasma IgA, IgM, IgG and ileal sIgA. We found that the *C. militaris* mycelium producing Mag II-CB treatment of the infected mice significantly improved the levels of plasma IgA, IgM, IgG and ileal sIgA (Fig. 8e–h). As shown in Fig. 8i, j, *E. coli* infection markedly increased the mRNA levels of IL-6 and TNF-α, but such increases were prevented by the *C. militaris* mycelium producing Mag II-CB. Moreover, the *C. militaris* mycelium producing Mag II-CB significantly increased expression of IL-10 cytokine (Fig. 8k). Overall, directly feeding with the *C. militaris* mycelium producing AMPs could protect mice from bacteria.

## Discussion

The routine treatment of livestock and poultry animals with antibiotics leaves unwanted traces of these agents in them, making the development of antibiotic alternatives as feed additives a worthwhile endeavor. Thus, AMPs with strong, broad spectrum antibacterial functions are promising candidates as feed additives. Previous studies have reported on the many types of expression systems available to produce recombinant AMPs. However, the edible and medicinal fungus, *C. militaris*, has not been reported as a vehicle for AMP production, despite its long-term use in disease prevention and treatment and its functional components, such as cordycepin and polysaccharides [37, 38]. Hence, in this study, we used *C. militaris* to produce the recombinant hybrid Mag II-CB antimicrobial peptide because of its potential positive effect on immune function and its ease of oral application. This is the first report of the successful expression of a recombinant antimicrobial peptide in *C. militaris*.

Our results showed that the recombinant hybrid Mag II-CB antimicrobial peptide had more obvious antibacterial activities than those of the single CB antimicrobial peptide, and a MIC between 2 and 32 μg/mL. These findings are consistent with previous studies, which have indicated that the antimicrobial peptide cecropin A (1-8)-magainin II (1-12) (CA-MA) had higher antibacterial activities than those of cecropin A or magainin II alone [27, 28, 39]. To further investigate the antibacterial activities of Mag II-CB and CB, we used mice infected with *E. coli* ATCC 25922 to determine the minimal lethal dose for this bacterium, and the dose of Mag II-CB and CB required to protect the mice from infection with it (Additional file 1: Additional materials). Several studies have shown that AMPs have a beneficial effect on the host by promoting intestinal health and maintaining the intestinal microbial balance [40, 41]. Our results have shown that the hybrid Mag II-CB antimicrobial peptide was highly effective at preventing the increase in *E. coli* numbers resulting from translocation of the bacteria after the intraperitoneal injection, as well as the decrease in *Lactobacillus* and *Bifidobacterium* numbers. This may be a superior feature of AMPs compared with traditional antibiotics, because several studies have indicated that traditional antibiotic treatments tend to decrease the intestinal flora, including probiotic bacteria such as *Lactobacillus* and *Bifidobacterium* [42]. The results of the in vitro and in vivo antibacterial assays indicate that the broad antimicrobial spectrum of the Mag II-CB hybrid antimicrobial peptide is probably related to the peptide fusion of each AMP.

AMPs protect hosts from bacterial infections via their direct antibacterial activities and their immunomodulatory functions. Immunoglobulin IgA, IgM and IgG are the innate immune effector molecules that can neutralize bacterial endotoxin and viral antigens by promoting the phagocytosis of monocytes and macrophages [43, 44]. Moreover, when sIgA is associated with intestinal lymphoid tissue, it can prevent the entry and adhesion of potentially harmful antigens in the intestine [45]. Shan et al. [46] reported that the antimicrobial peptide lactoferrin effectively increased serum IgA, IgM and IgG levels in weaning piglets. Wang et al. [47] also reported that AMPs could enhance sIgA expression levels in the intestines of specific pathogen-free chickens, which supports the notion that AMPs can improve intestinal mucosal immunity. However, interestingly, in the present study, we found that the experimental mice had significantly increased levels of plasma IgA, IgM, IgG and ileum sIgA after *E. coli* infection, which were reversed after treatment with the hybrid Mag II-CB antimicrobial peptide. We assume that this was because *E. coli* invasion stimulated the host innate immune response; however, the Mag II-CB treatment inhibited an excessive immune response in the mice, which implies that the recombinant Mag II-CB hybrid antimicrobial peptide can modulate immune function.

Because AMPs act as effector molecules in both innate and adaptive immunity, they can regulate pro-inflammatory and anti-inflammatory responses [36]. Under normal conditions, endotoxins derived from Gram-negative bacteria can only penetrate the intestinal epithelium in trace amounts because of the composite barrier effect of epithelial tight junctions, epithelial transcription factors and intestinal bacteria [48], however, when the intestinal permeability changes, pro-inflammatory cytokines are upregulated. As expected, we found that the hybrid Mag II-CB antimicrobial peptide protected the intestinal epithelial structure and intestinal permeability against endotoxin penetration and against the exogenous pathogen by increasing the integrity of the tight junctions. This also illustrates why Mag II-CB treatment reduced the mRNA levels of the pro-inflammatory cytokines IL-6, MCP-1 and TNF-α, and increased the expression of IL-10, an anti-inflammatory cytokine. Collectively, these results illustrate the significant impact of the recombinant AMPs on immunomodulatory functioning. Moreover, directly feeding the infected mice with the *C. militaris* mycelium producing AMPs further demonstrated that its antibacterial and immunomodulatory activities. The data presented here demonstrated the protective effects of AMPs in mice infected with *E. coli*. Recent studies have reported that a novel antibacterial peptide Cathlicidin-BF (C-BF) treatment may be a potential therapy for LPS/pathogen-induced intestinal injury in piglets [49]. And this provides us an effective therapeutic strategy, whether AMPs could exert the same therapeutic effects on post-infection model is in progress. And further studies will be conducted in the near future, including the underlying signaling pathway that regulates its immunomodulatory function.

## Conclusions

In summary, our findings suggest that AMPs from *C. militaris* mycelium display antibacterial and immunomodulatory activities in mice. The binary T-DNA expression vector used in this study could be utilized as a preliminary research tool for producing *C. militaris* transformants without selectable marker genes. Thus, with its well-known medicinal ingredients and the antibacterial and immunomodulatory effects of AMPs, transformed *C. militaris* has the potential to become a substitute to antibiotics as a feed additive for livestock.

## Methods

### Strains and growth conditions

The *E. coli* strain Trans5α (Transgen, China) and the *A. tumefaciens* strain EHA105 (Transduction Bio, China) were maintained in Luria–Bertani (LB) medium or YEP medium, respectively. *C. militaris* (provided by Jilin Agricultural University, China) was cultured on potato dextrose agar (PDA) plates. *E. coli* (ATCC 25922), *Staphylococcus aureus* (ATCC 25923), *Salmonella enteritidis*, *Bacillus subtilis*, *Enterococcus faecalis*, *Enterobacter aerogenes*, *Staphylococcus xylosus* and *Proteus mirabilis* were supplied by Jilin Agricultural University and maintained in LB medium or Mueller–Hinton (MH) broth.

### Construction of the pCB130-Mag II-CB and pCB130-CB expression vectors

The binary T-DNA expression vector pCB130-NG used in this study was provided by Jilin Agricultural University. The *Mag II*-*CB* and *CB* gene sequences were designed according the sequences of the mature Mag II and CB peptides in GenBank (Gene ID: J03193, D11114). A 6× His tag was inserted separately into the 5′ start region of the *Mag II*-*CB* or *CB* gene sequence. The codon choice was modified according to the codon usage of *C. militaris*. Each gene was synthesized by Genewiz (Jiang Su, China). The *Mag II*-*CB* and *CB* fragments obtained by digestion with *Xba*I and *Kpn*I were cloned individually into the pCB130-NG plasmid. Successful cloning was confirmed by PCR and restriction enzymatic analysis. Next, pCB130-Mag II-CB and pCB130-CB plasmids were separately transformed into *A. tumefaciens* EHA105 using the freeze–thaw method described previously [50].

### Tumefaciens-mediated transformation of *C. militaris* protoplasts

*Agrobacterium tumefaciens*-mediated protoplast transformation was conducted according to previous methodology [51, 52]. Briefly, the *A. tumefaciens* strain harboring the pCB130-Mag II-CB vector or the pCB130-CB vector was grown overnight to an optical density (OD)_600_ of 0.5–0.6 at 28 °C with shaking at 200 rpm in induction medium (IM). In parallel, a suspension containing 10^5^ *C. militaris* protoplasts/mL was extracted from WT *C. militaris* mycelium pellets using snail enzyme and lywallzyme. Co-cultures of *C. militaris* protoplasts and *A. tumefaciens*, which were spread onto 90-mm sterile filter papers laid on top of IM agar plates, were incubated at 25 °C for 2 days in the dark. Each filter paper was transferred to a selective PDA plate containing Hygromycin B (650 µg/mL) and cefotaxime (200 µg/mL), and then maintained at 25 °C in the dark until fungal colonies were visible. The *C. militaris* colonies obtained were cultured on PDA plates at 25 °C until three quarters of each plate contained mycelial growth.

### Molecular analysis of the transformants and purification and quantification of Mag II-CB and CB protein from *C. militaris*

Genomic DNA was extracted from the mycelia of the transformed *C. militaris* and the WT *C. militaris* using a Nuclean Plant Genomic DNA kit (CwBio, China). Successful transformation of the Mag II-CB and CB genes into *C. militaris* was determined by PCR analysis using the pCB130-specific primers (forward and reverse respectively): 5′-GCTCTAGAATGCATCATCATCACCACCAC-3′, and 5′-GGGGTACCTTAAATGGCCTTGGCGGAGC-3′. Protein extraction from WT *C. militaris* and transformed *C. militaris* mycelia was performed according to the method described previously [53]. Aliquots containing 50 μg of the proteins were loaded onto 16.5% Tricine-sodium dodecyl sulfate polyacrylamide gel electrophoresis (SDS-PAGE). After electrophoresis, the proteins were transferred to polyvinylidene difluoride (PVDF, 0.22 μm) membranes and then immunoblotted with a mouse primary antibody against the His tag (Transgen, China) and a secondary goat anti-mouse antibody (Transgen, China). Immunodetection was performed using a DAB kit (Solarbio, China).

His-tagged Mag II-CB and CB recombinant proteins were purified using Ni–NTA Sefinose resin His-tagged Columns (BBI Life Sciences, China). The protein extracts applied to the column were eluted with a solution containing 50 mM NaH_2_PO_4_, 300 mM NaCl, 300 mM Imidazole. The purified proteins were analyzed by Coomassie brilliant blue staining and western blotting. The concentrations of Mag II-CB and CB were determined using a Plant HIS-Tag enzyme-linked immunosorbent assay (ELISA) kit (R&D Systems, MN, USA), according to the manufacturer’s instructions. The plates were read at a wavelength of 450 nm.

### Antibacterial assays

Gram-negative and Gram-positive bacterial numbers were adjusted to 1 × 10^7^ colony forming units (CFU)/mL with MH broth and then spread onto MH agar plates for further use. Mag II-CB and CB were each added to the holes (8 mm in diameter) punched into the MH agar plates, and then cultured overnight at a temperature appropriate for each specific bacterium, after which the diameters of the inhibition zones were measured.

In parallel, MICs of Mag II-CB and CB were deter-mined by liquid growth inhibition assays. Mag II-CB and CB were individually diluted with sterile ddH_2_O to 128 μg/mL as stock solutions, and then serially diluted to different concentrations. Aliquots of each dilution together with the bacterial suspensions (5 × 10^5^ CFU/mL) were added to the wells of a 96-well polypropylene microtiter plate, which was incubated for 16 h on a 200 rpm shaker. The antibacterial activity was evaluated by measuring the peptide concentration responsible for 100% death of each bacterial pathogen at OD_600_.

### Mice and sample collection

#### I: effects of Mag II-CB and CB on BALB/c mice infected with *E. coli* (ATCC 25922)

Male BALB/c mice (Yisi, China) were divided into the following six groups: (1) normal control mice (NC); (2) mice infected with *E. coli* (ATCC 25922) (EI); (3) mice administered with recombinant Mag II-CB (MC, 8 mg/kg, 0.5 mL of Mag II-CB per 20 g); (4) mice administered with recombinant CB (CB, 8 mg/kg, 0.5 mL of CB per 20 g); (5) *E. coli*-infected mice administered with Mag II-CB (MC + EI); (6) *E. coli*-infected mice administered with CB (CB + EI). The NC and EI groups were intraperitoneally injected with saline for 5 days, whereas the other groups were injected with Mag II-CB or CB (dissolved in saline) using the same schedule. One hour after the last injection, the NC, MC, and CB groups were injected with saline and the other groups were injected with *E. coli* (0.5 mL/20 g, 10^9^ CFU/mL).

#### II: effects of the *C. militaris* mycelium producing AMPs on BALB/c mice infected with *E. coli* (ATCC 25922)

Male BALB/c mice (Yisi, China) were divided into the following eight groups: (1) normal control mice (NC); (2) mice infected with *Escherichia coli* (ATCC 25922) (EI); (3) mice administered with WT *C. militaris* mycelium powders (WT); (4) mice administered with the *C. militaris* producing Mag II-CB mycelium powders (MC); (5) mice administered with the *C. militaris* producing CB mycelium powders (CB); (6) *E. coli*-infected mice administered with WT *C. militaris* mycelium powders (WT + EI); (7) *E. coli*-infected mice administered with the *C. militaris* producing Mag II-CB mycelium powders (MC + EI); (8) *E. coli*-infected mice administered with the *C. militaris* producing CB mycelium powders (CB + EI). The freeze-dried mycelium powders mixed in the experimental diets and the concentration of Mag II-CB or CB in the final diets was 10 mg/kg. On the 5th day, the NC, WT, MC, and CB groups were injected with saline and the other groups were injected with *E. coli* (0.5 mL/20 g, 10^9^ CFU/mL).

All the mice were killed after 24 h. The mice were anesthetized with 4% chloral hydrate (0.1 mL/10 g), and their plasma and tissue samples were collected for analysis. All the mice were treated according to protocols reviewed and approved by the Institutional Animal Care and Use Committee of the Jilin Agricultural University.

### Biochemical assays and ileal secreted immunoglobulin A (sIgA) contents analysis

Plasma IgA, IgM and IgG were measured using mouse IgA, IgM and IgG assay ELISA kits (R&D Systems, MN, USA), respectively. Secreted immunoglobulin A (sIgA), which was extracted from the terminal ileum of each mouse, was measured using a mouse sIgA ELISA kit (R&D Systems, MN, USA). ELISAs were performed according to the manufacturer’s manual. The plates were read at a wavelength of 450 nm.

### Intestinal microbial flora assay and intestinal histology

The numbers of *E. coli* and probiotic bacteria (*Lactobacillus* and *Bifidobacterium*) in the cecal contents of each mouse were counted using the dilution plate colony counting method. Briefly, cecal contents were homogenized and 100 µL of each gradient-diluted suspension (10^2^–10^4^-fold) was plated onto the selective plates. All the plates were maintained at 37 °C for 18–24 h.

Each 10% formalin-fixed and paraffin-embedded ileum section of 5 µm thickness was stained with hematoxylin and eosin, and then analyzed by light microscopy. The villus length, crypt depth and V/C value (villus length/crypt depth) were also recorded.

### Quantitative real-time reverse transcriptase (RT)-PCR

In brief, total RNA isolated using RNAiso Plus reagent (Takara, Japan) was reverse transcribed into cDNA obtained using the PrimeScript™ RT reagent Kit with gDNA Eraser (Takara, Japan). cDNA amplification was performed in 96-well reaction plates with a SYBR Premix Ex Taq Kit (Takara, Japan). Primer sequences (forward and reverse respectively) were as follows: ZO-1 5′-TGGGAACAGCACACAGTGAC-3′, and 5′-GCTGGCCCTCCTTTTAACAC-3′; Claudin-1 5′-CGGGCAGATACAGTGCAAAG-3′, and 5′-ACTTCATGCCAATGGTGGAC-3′; Occludin 5′-ACCCGAAGAAAGATGGATCG-3′, and 5′-CATAGTCAGATGGGGGTGGA-3′; IL-6 5′-GAGTCACAGAAGGAGTGGCTAAGGA-3′, and 5′-CGCACTAGGTTTGCCGAGTAGATCT-3′; MCP-1 5′-CCCACTCACCTGCTGCTACTCATT-3′, and 5′-CTACAGCTTCTTTGGGACACCTGCT-3′; TNF-α 5′-AGGGGACATTCCTGTGTTCC-3′, and 5′-TTACCCTGTTTCCCCATTCC-3′; IL-10 5′-GGACCAGCTGGACAACATACTGCTA-3′, and 5′-CCGATAAGGCTTGGCAACCCAAGT-3′; β-actin 5′-GAGACCTTCAACACCCC-3′, and 5′-ATAGCTCTTCTCCAGGGAGG-3′. The relative quantities of the target transcripts were analyzed from duplicate samples after normalization against β-actin, an endogenous reference gene. The specificity of the primers was confirmed by dissociation curve analysis after PCR amplification. The relative mRNA expression level was quantified using the ΔΔCt method.

### Statistical analysis

All the experiments were repeated at least three times. ANOVA and the Newman–Keuls multiple-comparison test were used to test for statistical significance. Differences between the groups were considered statistically significant when P < 0.05. The data presented are the mean values ± the standard errors.

## Additional file

**Additional file 1: Figure S1.** PCR analysis of the Mag II-CB (**a**) and CB (**b**) gene in wild-type and transformed *C. militaris*. The PCR products were analyzed on a 1.0% (w/v) agarose gel. Lane 1: bank control (ddH_2_O). Lane 2: negative control (WT *C. militaris*). Lane 3: positive control (plasmid pCB130-Mag II-CB or pCB130-CB). Lanes 4–15 (**a**): PCR products generated from Mag II-CB transformants. Lanes 4–18 (**b**): PCR products generated from CB transformants. M, 2000-bp DNA ladder. **Table S1.** Determining the minimal lethal dose (MLD) for *E. coli* (ATCC 25922) in mice. **Table S2.** Determining the concentration of the recombinant peptide needed for antibacterial protection.
