# Supplementary material for: Expression of a recombinant hybrid antimicrobial peptide magainin II-cecropin B in the mycelium of the medicinal fungus Cordyceps militaris and its validation in mice
Source: Microb Cell Fact. 2018 Feb 5;17:18. doi: 10.1186/s12934-018-0865-3 (PMC5798188; doi:10.1186/s12934-018-0865-3)
Supplement: Supplementary file 1 — Additional file 1: Figure S1. PCR analysis of the Mag II-CB (a) and CB (b) gene in wild-type and transformed C. militaris. The PCR products were analyzed on a 1.0% (w/v) agarose gel. Lane 1: bank control (ddH2O). Lane 2: negative control (WT C. militaris). Lane 3: positive control (plasmid pCB130-Mag II-CB or pCB130-CB). Lanes 4–15 (a): PCR products generated from Mag II-CB transformants. Lanes 4–18 (b): PCR products generated from CB transformants. M, 2000-bp DNA ladder. Table S1. Determining the minimal lethal dose (MLD) for E. coli (ATCC 25922) in mice. Table S2. Determining the concentration of the recombinant peptide needed for antibacterial protection. [file 12934_2018_865_MOESM1_ESM.doc]

Additional materials: Expression of a recombinant hybrid antimicrobial peptide magainin II-cecropin B in the mycelium of the medicinal fungus *Cordyceps militaris* and its validation in mice

Min Zhang, Yuan-Long Shan, Hong-Tao Gao, Bin Wang, Xin Liu, Yuan-Yuan Dong, Xiu-Ming Liu, Na Yao, Yong-Gang Zhou, Xiao-Wei Li * and Hai-Yan Li *

Methods

To determine the minimal lethal dose (MLD) for *E. coli* (ATCC 25922) in mice, male BALB/c mice were divided into three groups (n =6 per group) and injected with different doses of *E. coli* (0.5 mL per 20 g) before the protection experiments. The number of deaths in mice was observed and recorded within 24 h.

Next, we determined the concentration of the recombinant peptides needed for antibacterial protection. Male BALB/c mice were divided into eight groups (n =6 per group): 1) mice administered with recombinant Mag II-CB (2 mg/kg, 0.5 mL of Mag II-CB per 20 g); 2) mice administered with recombinant CB (2 mg/kg, 0.5 mL of CB per 20 g); 3) mice administered with recombinant Mag II-CB (4 mg/kg, 0.5 mL of Mag II-CB per 20 g); 4) mice administered with recombinant CB (4 mg/kg, 0.5 mL of CB per 20 g); 5) mice administered with recombinant Mag II-CB (6 mg/kg, 0.5 mL of Mag II-CB per 20 g); 6) mice administered with recombinant CB (6 mg/kg, 0.5 mL of CB per 20 g); 7) mice administered with recombinant Mag II-CB (8 mg/kg, 0.5 mL of Mag II-CB per 20 g); 8) mice administered with recombinant CB (8 mg/kg, 0.5 mL of CB per 20 g). All the mice were injected with a MLD dose (1×10^9^ CFU/mL) of *E. coli.* The number of deaths in mice was observed and recorded within 24 h.

**Table S1. Determining the minimal lethal dose (MLD) for *E. coli* (ATCC 25922) in mice**

| **Group** | **Number of mice** | **Injection dose (CFU/mL)** | **Number of deaths** | **Mortality (%)** |
| --- | --- | --- | --- | --- |
| A | 6 | 5×10^8^ | 3 | 50 |
| B | 6 | 1×10^9^ | 6 | 100 |
| C | 6 | 2×10^9^ | 6 | 100 |

A, B, and C represent groups injected with different doses of *E. coli* (ATCC 25922).

**Table S2.** Determining the concentration of the recombinant peptide needed for antibacterial protection

| **Group** | **Number of mice** | **Antimicrobial peptide dose (mg/kg)** | **Number of deaths** | |
| --- | --- | --- | --- | --- |
|  |  |  | Mag II-CB | CB |
| A | 6 | 2 | 6 | 6 |
| B | 6 | 4 | 6 | 6 |
| C | 6 | 6 | 2 | 3 |
| D | 6 | 8 | 0 | 0 |

A, B, C and D represent groups injected with different doses of recombinant antimicrobial peptide.

**
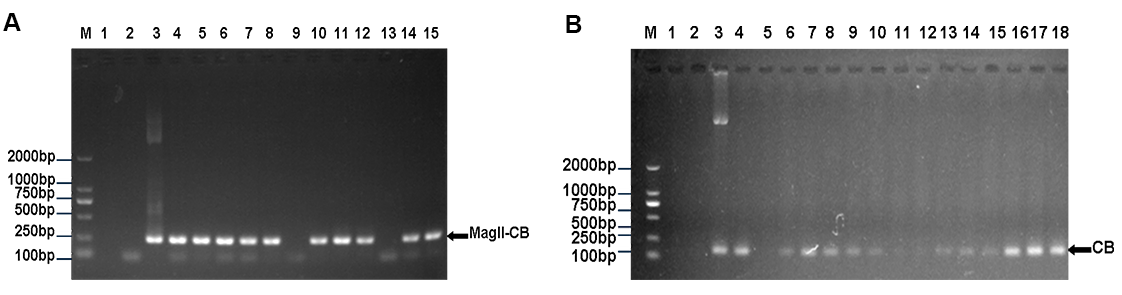
**

**Figure S1.** PCR analysis of the Mag II-CB (**A**) and CB (**B**) gene in wild-type and transformed *C. militaris*. The PCR products were analyzed on a 1.0% (w/v) agarose gel. Lane 1: bank control (ddH_2_O). Lane 2: negative control (WT *C. militaris*). Lane 3: positive control (plasmid pCB130-Mag II-CB or pCB130-CB). Lanes 4–15 (panel **A**): PCR products generated from Mag II-CB transformants. Lanes 4–18 (panel **B**): PCR products generated from CB transformants. M, 2000-bp DNA ladder.
